# Aging Triggers Mitochondrial Dysfunction in Mice

**DOI:** 10.3390/ijms241310591

**Published:** 2023-06-24

**Authors:** Frederico Luis Lima Rosa, Itanna Isis Araujo de Souza, Gustavo Monnerat, Antonio Carlos Campos de Carvalho, Leonardo Maciel

**Affiliations:** 1 Instituto de Biofísica Carlos Chagas Filho, Universidade Federal do Rio de Janeiro, Rio de Janeiro 21941-902, RJ, Brazil; fredllr@gmail.com (F.L.L.R.); itanna.isis258@gmail.com (I.I.A.d.S.); monnerat1988@gmail.com (G.M.); acarlos@biof.ufrj.br (A.C.C.d.C.); 2 Instituto Nacional de Ciência e Tecnologia em Medicina Regenerativa, Rio de Janeiro 21941-902, RJ, Brazil; 3 Campus Professor Geraldo Cidade, Universidade Federal do Rio de Janeiro, Duque de Caxias 25240-005, RJ, Brazil

**Keywords:** aging, heart, mitochondrial dysfunction

## Abstract

Direct analysis of isolated mitochondria from old mice enables a better understanding of heart senescence dysfunction. Despite a well-defined senescent phenotype in cardiomyocytes, the mitochondrial state in aged cardiomyocytes is still unclear. Here, we report data about mitochondrial function in old mice. Isolated cardiomyocytes’ mitochondria were obtained by differential centrifugation from old and young mice hearts to perform functional analyses of mitochondrial O_2_ consumption, transmembrane potential, ROS formation, ATP production, and swelling. Our results show that mitochondria from old mouse hearts have reduced oxygen consumption during the phosphorylative states of complexes I and II. Additionally, these mitochondria produced more ROS and less ATP than those of young hearts. Mitochondria from old hearts also showed a depolarized membrane potential than mitochondria from young hearts and, as expected, a greater electron leak. Our results indicate that mitochondria from senescent cardiomyocytes are less efficient in O_2_ consumption, generating more ROS and producing less ATP. Furthermore, the phosphorylative state of complexes I and II presents a functional defect, contributing to greater leakage of protons and ROS production that can be harmful to the cell.

## 1. Introduction

Aging is an inevitable biological ongoing process of all the higher organisms [1]. Although the aging process has not been entirely deciphered, pro-aging mechanisms occur through deoxyribonucleic acid (DNA) damage, peroxidation, and protein misfolding, resulting in cell death or senescence [2,3,4].

The impairment of mitochondrial function and morphology has been correlated with aging, cell senescence, and aging-related pathological disorders, including heart disease [5,6,7]. An increase in reactive oxygen species (ROS) production due to mitochondrial dysfunction is characteristic in several models of cellular senescence, including stress-induced senescence [8], replicative senescence [9], oncogene-induced senescence [10], and senescence triggered by genetic telomere uncapping [11]. Apparently, the high production of ROS is a characteristic of aging and senescent cells, being mechanistically responsible for the progression of senescence and cellular damage [12]. Besides increased ROS production, mitochondria of aged or senescent cells show genomic instability, decompensated mitochondrial turnover, unbalanced NAD^+^/NADH ratio, and a Ca^2+^ overload [13].

Heart mitochondria and aging have been the subject of extensive research due to the crucial role of mitochondria in cardiac function and the impact of aging on cardiovascular health [14]. Apparently, there are age-related changes in mitochondrial structure and function in the heart, with alterations in mitochondrial morphology, increasing oxidative stress [14], contributing to the decline in cardiac function during aging [14]. In addition, it seems to have an age-dependent increase in the mitochondrial DNA mutations in the hearts. These mutations can impair mitochondrial function and contribute to the age-related decline in cardiac performance [15]. Furthermore, the decrease in molecular mechanisms of mitochondrial quality control, such as mitophagy, has been correlated to heart aging. Once the aged heart has shown reduced mitophagy activity, the accumulation of damaged mitochondria can exacerbate mitochondrial dysfunction and contribute to age-related cardiac pathologies [15].

Despite the well-established link between aging, cellular senescence, and mitochondrial dysfunction, our understanding of the mitochondrial characteristics of cells from aged animals remains limited [5,6,7,13]. Therefore, the primary objective of this study was to investigate the functional phenotype of cardiac mitochondria derived from aged animals and compare these findings with the mitochondrial phenotype observed in the hearts of young animals.

## 2. Results

### 2.1. Mitochondrial Respiration

**Figure 1:**
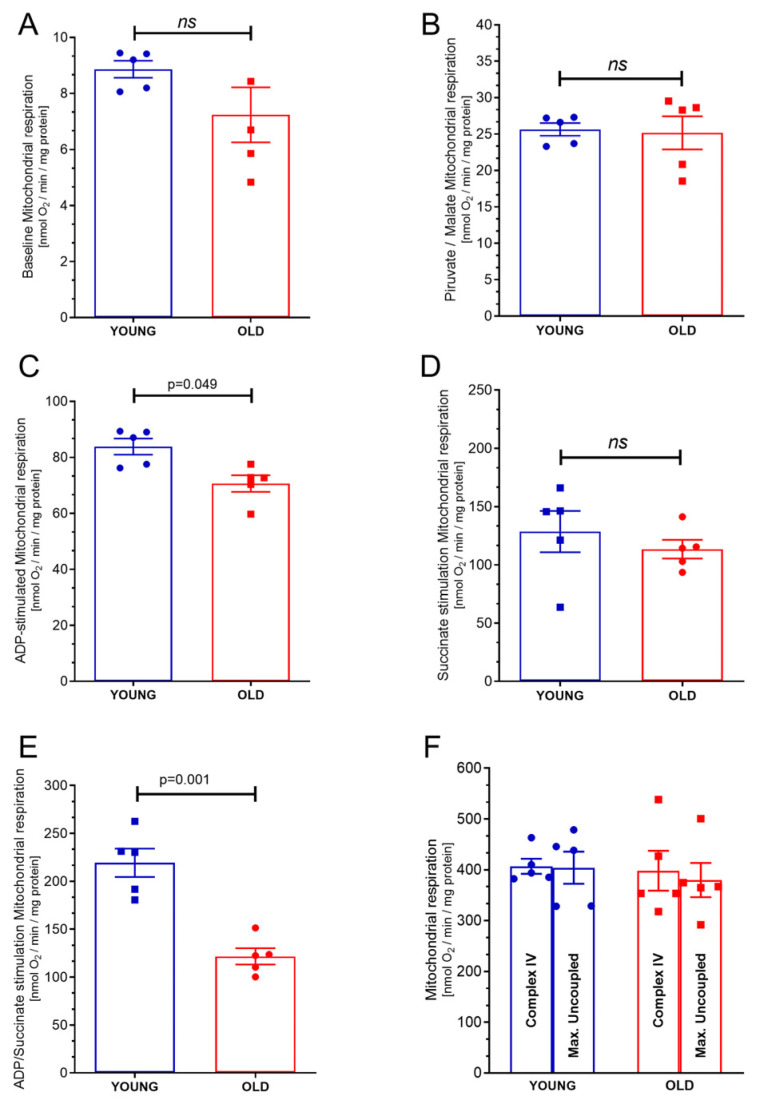
Respiration of isolated mitochondria from young and old hearts: (**A**) Baseline respiration (state 1 complex 1); (**B**) pyruvate/malate stimulation (state 2 complex 1) respiration; (**C**) adenosine diphosphate (ADP) stimulation (state 3 complex 1) respiration. (**D**) Complex II under state 2 respiration was stimulated by succinate and using the complex I inhibitor Rotenone. (**E**) Complex II under state 3 respiration was stimulated by succinate plus ADP and using the complex I inhibitor Rotenone. (**F**) Complex IV respiration stimulated by N,N,N,N-Tetramethyl-p-phenylenediamine dihydrochloride (TMPD) and ascorbate and maximal uncoupled oxygen uptake induced by carbonyl cyanide 4-(trifluoromethoxy)phenylhydrazone (FCCP). Each symbol represents one animal. The values are reported as mean ± S.E.M. Horizontal square brackets indicate significantly different differences and the corresponding *p*-value.

The data presented in Figure 1A illustrate that there was a resemblance in the mitochondrial oxygen consumption by complex I under state 1 between mitochondria obtained from the hearts of young mice (8.86 ± 0.30 nmol O_2_/min/mg protein) and those from old mice (7.23 ± 0.98 nmol O_2_/min/mg protein, *p* = 0.19). These findings suggest that there was no significant difference in the rates of oxygen consumption observed in the mitochondria from the two groups, implying that age did not have a notable effect on complex I under state 2 oxygen consumption.

According to the data depicted in Figure 1B, it is evident that there was no significant difference (*p* = 0.99) in the mitochondrial oxygen consumption by complex I under state 2 when comparing mitochondria derived from the hearts of young mice (25.63 ± 0.87 nmol O_2_/min/mg protein) to those obtained from the hearts of old mice (25.7 ± 4.7 nmol O_2_/min/mg protein, *p* = 0.855). These findings suggest that, regardless of age, the oxygen consumption rates of complex I under state 2 remained relatively consistent in the mitochondria derived from mice hearts, highlighting a lack of substantial variation in complex I under state 2 oxygen consumption between the two groups.

The data presented in Figure 1C indicate that there was a notable decrease in the mitochondrial oxygen consumption by complex I under state 3 in mitochondria derived from the hearts of old mice (70.81 ± 3.1 nmol O_2_/min/mg protein) compared to mitochondria obtained from young mice hearts (78.81 ± 2.8 nmol O_2_/min/mg protein, *p* = 0.049). These findings suggest that age may have contributed to a reduction in the oxygen consumption rates of complex I under state 3 in the mitochondria obtained from mice hearts.

According to the data depicted in Figure 1D, it is evident that there was no significant difference in the mitochondrial oxygen consumption by complex II under state 2 between mitochondria derived from young mice hearts (128.6 ± 17.73 nmol O_2_/min/mg protein) and those obtained from old mice hearts (113.5 ± 8.0 nmol O_2_/min/mg protein, *p* = 0.38). This observation suggests that the ability of complex II to utilize oxygen under state 2 remained relatively consistent across both age groups, as visually depicted in the figure.

However, a contrasting pattern emerged when investigating the mitochondrial oxygen consumption by complex II under state 3 (phosphorylative state), as shown in Figure 1E. Notably, mitochondria obtained from old mice hearts (121.14 ± 8.55 nmol O_2/_min/mg protein) exhibited a reduced oxygen consumption rate compared to mitochondria derived from young mice hearts (218.3 ± 14.84 nmol O_2/_min/mg protein, *p* = 0.0013). These findings suggest that the phosphorylative state of complex II was affected by age, demonstrating a decline in mitochondrial oxygen consumption in the hearts of older mice, as depicted in Figure 1E.

As depicted in the Figure 1F, the mitochondrial oxygen consumption by maximal oxygen uptake of uncoupled mitochondria exhibited similarities between mitochondria obtained from young mice hearts (460.5 ± 14.8 nmol O_2/_min/mg protein) and those derived from old mice hearts (398.01 ± 39.2 nmol O_2_/min/mg protein, *p* = 0.8). Furthermore, the mitochondrial oxygen consumption by complex IV demonstrated resemblances when comparing mitochondria derived from young mice hearts (403.2 ± 31.2 nmol O_2_/min/mg protein) to those obtained from old mice hearts (379.71 ± 33.9 nmol O_2_/min/mg protein, *p* = 0.7). These findings imply that there were no significant differences in mitochondrial loading (complex IV), and the mitochondria show similar viability (maximal uncoupled oxygen uptake) between the two age groups, as supported by the provided data.

### 2.2. Mitochondrial ROS Production

**Figure 2:**
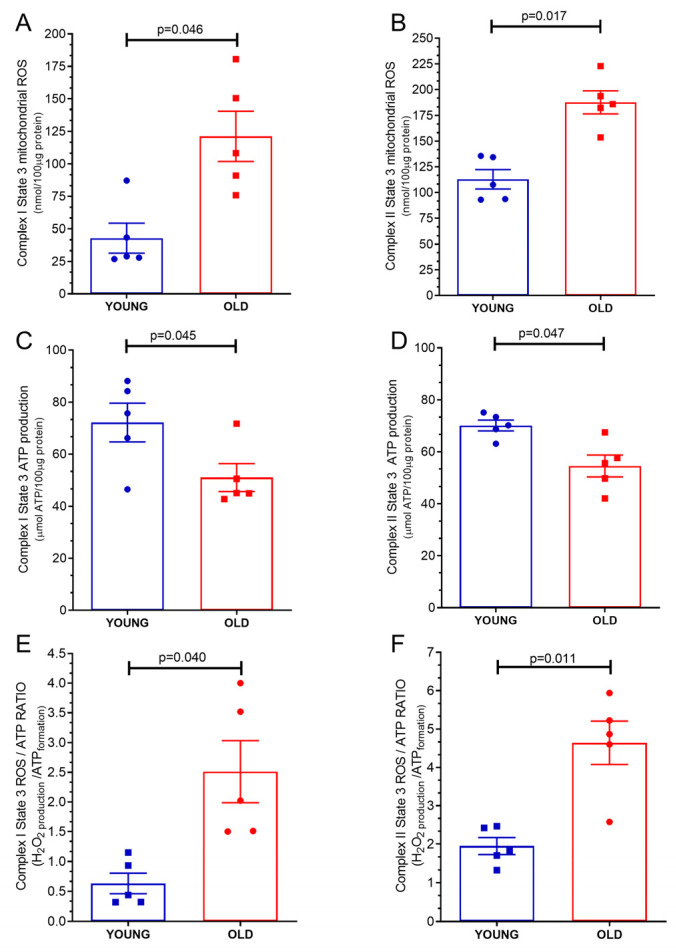
Mitochondrial products and characteristics of isolated mitochondria from young and old hearts: (**A**) reactive oxygen species (ROS) production under state 3 complex I stimulation; (**B**) reactive oxygen species (ROS) production under state 3 complex II stimulation; (**C**) adenosine triphosphate (ATP) production under state 3 complex I stimulation; (**D**) adenosine triphosphate (ATP) production under state 3 complex II stimulation, (**E**) ROS/ATP ratio under state 3 complex I stimulation; (**F**) ROS/ATP ratio under state 3 complex II stimulation. Each symbol represents one animal. Data are expressed as mean ± S.E.M. Horizontal square brackets indicate significantly different differences and the corresponding *p*-value.

As illustrated in Figure 2A, the mitochondrial ROS production under complex I state 3 stimulation (phosphorilative state) exhibited an elevation in mitochondria obtained from the hearts of old mice (42.84 ± 11.47 nmol/loaded μg protein) compared to mitochondria derived from young mice hearts (121.29 ± 19.38 nmol/loaded protein, *p* = 0.046). These findings suggest that age may have contributed to an increase in mitochondrial ROS production during complex I state 3 stimulation. Similarly, Figure 2B demonstrates that the mitochondrial ROS production under complex II state 3 (phosphorilative state) was higher in mitochondria derived from the hearts of old mice (112.9 ± 9.4 nmol/loaded μg protein) compared to mitochondria obtained from young mice hearts (187.7 ± 11.11 nmol/loaded μg protein, *p* = 0.0176). This observation suggests that aging might be associated with an elevation in mitochondrial ROS production during complex II state 3 stimulation.

### 2.3. Mitochondrial Adenosine Triphosphate (ATP) Production

As visually depicted in Figure 2C, the mitochondrial ATP production under complex I state 3 stimulation (phosphorilative state) exhibited a decrease in mitochondria obtained from the hearts of old mice (51.01 ± 5.33 μmol ATP/loaded protein) compared to mitochondria derived from young mice hearts (72.16 ± 7.4 μmol ATP/loaded protein, *p* = 0.045). These findings suggest that age may have contributed to a reduction in mitochondrial ATP production during complex I state 3 stimulation, therefore, reducing the energy availability of the tissue. Similarly, Figure 2D illustrates that the mitochondrial ATP production under complex II state 3 stimulation (phosphorilative state) was lower in mitochondria derived from the hearts of old mice (54.48 ± 4.21 μmol ATP/loaded protein) compared to mitochondria obtained from young mice hearts (70.10 ± 2.0 μmol ATP/loaded protein, *p* = 0.047). This observation suggests that aging may be associated with a decrease in mitochondrial ATP production during complex II state 3 stimulation and that this low availability of ATP is probably due to a malfunction of complexes I and II during the phosphorylative state.

### 2.4. Mitochondrial ATP/ROS Ratio

The mitochondrial ATP/ROS ratio from old mice hearts (0.63 ± 0.17 ATP formation/H_2_O_2_ production) was lower than mitochondria from young mice hearts (2.51 ± 0.52 ATP formation/H_2_O_2_ production, *p* = 0.04), under complex I state 3 stimulation (phosphorilative state), as shown in Figure 2E. The mitochondrial ATP/ROS ratio from old mice hearts (1.94 ± 0.21 ATP formation/H_2_O_2_ production), under complex II state 3 stimulation, was also lower than mitochondria from young mice hearts (4.60 ± 0.56 ATP formation/H_2_O_2_ production *p* = 0.013), as depicted in Figure 2F.

### 2.5. Mitochondrial Transmembrane Potential Δψ

**Figure 3:**
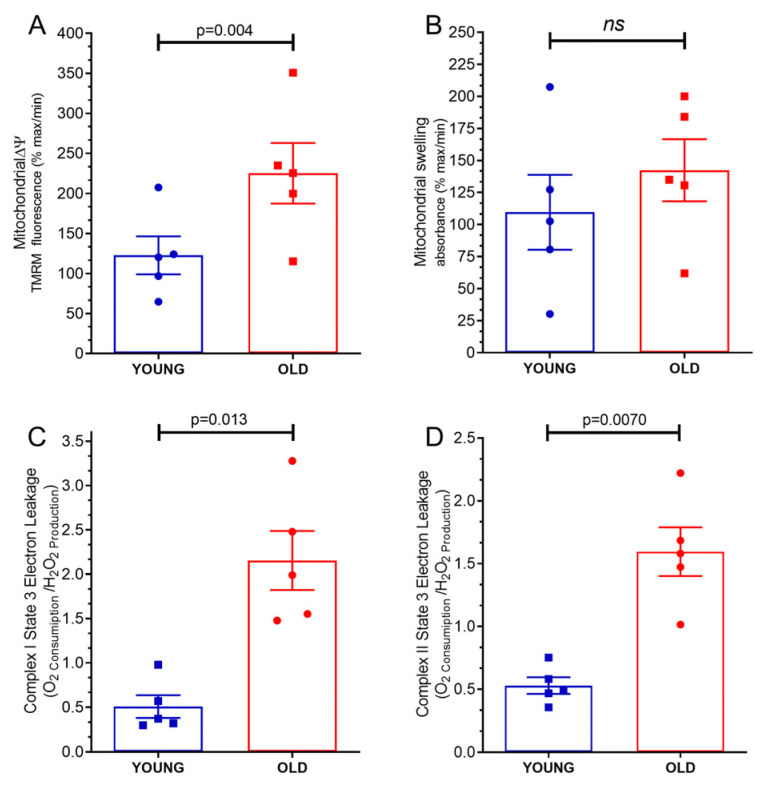
Characteristics of isolated mitochondria from young and old hearts: (**A**) mitochondrial transmembrane potential (mΔψ); (**B**) mitochondrial swelling; (**C**) electron leakage under state 3 complex I stimulation; (**D**) electron leakage under state 3 complex II stimulation. Each symbol represents one animal. Data are expressed as mean ± S.E.M. Horizontal square brackets indicate significantly different differences and the corresponding *p*-value.

The study yielded compelling results highlighting a significant disparity in the mitochondrial transmembrane potential Δψ between mitochondria derived from the hearts of old mice and those obtained from young mice. As illustrated in Figure 3A, the measurement of mitochondrial transmembrane potential Δψ in old mice hearts recorded a higher value (225.1 ± 37.82% maximum) compared to the measurement in mitochondria from young mice hearts, which exhibited a lower value (122.6 ± 23.67% maximum). Notably, the observed difference between these two measurements was deemed statistically significant, supported by a *p*-value of 0.004. The mitochondrial transmembrane potential Δψ holds critical importance in cellular energetics and function. It refers to the electric potential difference across the inner mitochondrial membrane, serving as a driving force for ATP synthesis during oxidative phosphorylation. A depolarized mitochondrial transmembrane potential Δψ is indicative of an inefficient mitochondrial energy production system.

### 2.6. Mitochondrial Swelling

The measurement of mitochondrial swelling in old mice hearts was (142.3 ± 22.22% maximum), while the measurement in mitochondria from young mice hearts was (109.6 ± 29.20% maximum), as shown in Figure 3B. However, the results of this study showed that there was no significant difference in swelling between mitochondria from old mice hearts and mitochondria from young mice hearts (*p* = 0.41).

### 2.7. Mitochondrial Proton Leakage

As demonstrated in Figure 3C, the analysis revealed that there was a noticeable increase in mitochondrial proton leakage in the mitochondria from old mice hearts (2.15 ± 0.33 H_2_O_2_ Production/O_2_ Consumption) as compared to mitochondria from young mice hearts (0.50 ± 0.12 H_2_O_2_ Production/O_2_ Consumption) under the complex I state 3 stimulation (phosphorilative state, *p* = 0.013). Moreover, it was observed that the mitochondrial proton leakage in the old mice hearts was also higher than mitochondrial proton leakage in young mice hearts when subjected to complex II state 3 stimulation, phosphorilative state (1.59 ± 0.19 H_2_O_2_ Production/O_2_ Consumption, *p* = 0.0070).

## 3. Discussion

The current study sought to investigate the effects of aging on cardiac mitochondrial function by examining various parameters of mitochondrial respiration, ROS production, ATP production, mitochondrial membrane potential, mitochondrial swelling, and proton leakage. The findings of this study suggest that the aging process has a significant impact on cardiac mitochondrial function.

One of the most important findings of this study was that cardiac mitochondrial oxygen consumption was significantly lower in old mice than in young mice. Besides showing a slight reduction in oxygen consumption by complex I under the phosphorylative state (state 3), we observed a strong reduction in oxygen consumption by complex II state 3. This finding suggests that aging reduces mitochondrial respiratory capacity. Furthermore, this dysfunction in the complex II state 3 may indicate a characteristic failure of the phosphorylative system during aging. The decreased respiratory capacity in old mice could be attributed to an age-related decline in the expression and activity of electron transport chain complexes or a decrease in the number of functional mitochondria [16,17].

Additionally, our study demonstrated that under complex I and complex II state 3 stimulation, the production of mitochondrial ROS was higher in the hearts of older animals than in younger animals. This finding raises the possibility that the age-related decline in the antioxidant defense system [18], which lead to results in oxidative stress and cellular damage and may be the cause of the increased ROS production [16]. These results are in agreement with the previously published studies that describe transcriptional changes in pathways related to ROS in the heart [18,19]. Our study also showed that the mitochondrial ATP production under stimulation of complex I and complex II state 3 was significantly lower in the hearts of old versus young mice. This finding suggests that the production of mitochondrial ATP declines with age. The reduction in the number of functional mitochondria or the age-related decline in the activity of the electron transport chain complexes, which produce the proton gradient for ATP synthesis, may be the cause of this decline in ATP production [20].

Interesting data, in particular, are the significantly depolarized mitochondrial membrane potential in the hearts of old versus young mice. This result explains the increase in mitochondrial ROS production in old mice once a membrane depolarization reduces the rate of electron flow, increasing the degree of electronic reduction in the phosphorylative chain and generating more ROS [21]. Additionally, it explains the reduction in the production of ATP because the membrane depolarization also reduces the H+ gradient, reducing the H^+^ driving force through the ATP-synthase and impairing its functioning [22,23]. The age-related changes in the expression and activity of the electron transport chain complexes or the mitochondrial uncoupling proteins may be the cause of the elevated mitochondrial membrane potential [24]. Finally, the study showed that under complex I and II state 3 stimulation, the mitochondrial proton leakage was significantly higher in old mice compared to young mice hearts. This finding indicates that the mitochondrial proton leakage increases with aging, possibly as a result of the aging-related decline in activity of the electron transport chain complexes or the mitochondrial uncoupling proteins [25]. Interestingly, a recent study using induced pluripotent stem cell-derived cardiomyocytes from a patient with Hutchinson–Gilford Progeria Syndrome found that premature aging is also associated with mitochondrial alterations in cardiac cells. The study discovered structural abnormalities in mitochondria, while high-resolution proteomics data revealed significant changes in protein expression related to the tricarboxylic acid cycle [26].

Some limitations should be considered when interpreting the findings of the present study. Firstly, the sample size of this study was relatively small, which may have limited the statistical power to detect the significant differences between groups. Second, we only looked at mitochondrial function in heart tissue, so the findings may not be generalizable to other tissues or organs. Finally, the study did not investigate all the underlying molecular mechanisms of the observed changes in mitochondrial function, and more research is needed to elucidate the underlying molecular mechanisms. Despite these limitations, the current study provides important information on age-related changes in mitochondrial function, which may have implications for the development of interventions to prevent age-related diseases.

## 4. Materials and Methods

### 4.1. Materials

All chemicals (analytical grade) were obtained from Sigma-Aldrich (San Luis, MO, USA) if not otherwise specified. All solutions were freshly prepared and filtrated (1.2 μm filter, Millipore, Burlington, MA, USA).

### 4.2. Animals

This study conformed to the Guide for the Care and Use of Laboratory Animals published by the US National Institute of Health (8th edition, 2011), and the experimental protocols were approved by the local Institutional Animal Care and Use Committee (protocol 01200.001568/2013-87). The animals were maintained in an animal room with controlled light (12:12-h light–dark cycle) and temperature (23–24 °C). The animals had ad libitum access to food and water. In this study, 5 male animals per group were used, totaling 10 animals. The animals of the YOUNG group are C57BL/6J mice at 16 weeks± 5 days of age. The animals of the OLD group are C57BL/6J mice at 104 weeks ± 3 days of age (aged mice were acquired at 78 weeks ± 3 days, IMSR_JAX:000664 from Jackson Laboratory, Bar Harbor, ME, USA).

### 4.3. Mitochondria Isolation and Measurement of Mitochondrial Function

The process of isolating mitochondria was conducted in accordance with previously established protocols [21]. Mice were euthanized, a thoracotomy was performed, the heart was carefully removed and immediately placed in a tube containing an ice isolation buffer at 4 °C (250 mmol·L^−1^ of sucrose; 10 mmol·L^−1^ of HEPES; and 1 mmol·L^−1^ of ethylene glycol tetra-acetic acid (EGTA). The pH was adjusted to 7.4 using TRIS solution) to remove excess blood. In order to enhance the process, 0.5% *w*/*v* bovine serum albumin (BSA) was incorporated into the buffer. All adipose tissue was removed using scissors. The tissue was minced into small pieces using scissors until the size of the tissue was about 1–2 mm and all fat that remained was removed, followed by homogenization utilizing a tissue homogenizer Ultra-Turrax. The samples were subjected to two 10 s cycles at a rotation rate of 6500 rounds per minute on ice. The samples were further homogenized using a tissue glass Potter–Elvehjem homogenizer and performed with the aid of proteinase type XXIV (8 IU/mg tissue weight) using a Teflon pestle. The resultant homogenate underwent centrifugation at 700× *g* for a duration of 10 min at a temperature of 4 °C. Subsequently, the supernatant was carefully collected and subjected to another round of centrifugation at 12,300× *g* for 10 min. The resulting pellet was resuspended in an ice-cold isolation buffer, omitting the presence of BSA, and underwent centrifugation once again at 10,300× *g* for 10 min at 4 °C. This sequential process was repeated, and the final pellet was resuspended in an isolation buffer. The concentration of protein within the isolated pellet was determined using a protein assay, specifically the Lowry method provided by Biorad (Hercules, CA, USA), with a BSA standard (Thermo Scientific, Waltham, MA, USA) serving as the comparative reference.

### 4.4. Mitochondrial Oxygen Consumption

Here, mitochondrial complex I (state 1, 2, and 3), II (state 1, 2, and 3), and IV respiration with subsequent uncoupling of oxidative phosphorylation were measured in a two-chamber respirometer.

Mitochondrial respiration was measured with a Clark-type electrode (Strathkelvin, Glasgow, UK) at 37 °C during magnetic stirring in incubation buffer containing 125 mmol·L^−1^ of KCl; 10 mmol·L^−1^ of MOPS; 2 mmol·L^−1^ of MgCl_2_; 5 mmol·L^−1^ of KH_2_PO_4_; and 0.2 mmol·L^−1^ of EGTA with glutamate (5 mmol·L^−1^) and malate (5 mmol·L^−1^) as substrates for complex I or succinate (5 mmol·L^−1^) as substrates for complex II. The oxygen electrode was calibrated using a solubility coefficient of 217 nmol O_2_/mL at 37 °C. For the measurement of complex I respiration, mitochondria (corresponding to a mitochondrial protein amount of 50 µg) was added to 1 mL incubation buffer. After 2 min of incubation, 1 mmol·L^−1^ ADP was added, and ADP-stimulated respiration was measured for 2 min. For the measurement of complex II respiration, the complex I inhibitor Rotenone (1 µmol·L^−1^) was added. Afterward, mitochondria were used to either measure complex IV respiration and maximal uncoupled oxygen uptake in the respiration chamber, or an incubation buffer containing mitochondria was taken from the respiration chamber to measure ATP production or mitochondrial ROS concentration, respectively. Complex IV respiration was stimulated by adding N,N,N,N′-tetramethyl-p-phenylenediamine (TMPD, 300 μmol·L^−1^) plus ascorbate (3 μmol·L^−1^), which donates electrons to cytochrome oxidase via the reduction in cytochrome c. Maximal uncoupled oxygen uptake was measured in the presence of 30 nmol·L^−1^ carbonyl cyanide-p-trifluoromethoxyphenyl-hydrazone (FCCP) [23,27,28].

### 4.5. Mitochondrial ATP Production

After the measurement of ADP-stimulated respiration of complex I and complex II, the incubation buffer containing mitochondria was taken from the respiration chamber and immediately supplemented with the ATP assay mix diluted 1:5 (ATP Bioluminescence Assay Kit, Sigma-Aldrich, St. Louis, MO, USA). Mitochondrial ATP production was determined immediately after each respiration measurement and compared with ATP standards using a 96-well white plate in a spectrofluorometer (SpectraMax^®^ M3, Molecular Devices, San Jose, CA, USA) at 560-nm emission [23,27,28].

### 4.6. Mitochondrial Swelling and Transmembrane Potential

The mitochondrial swelling and transmembrane potential were evaluated using a spectrofluorometer (SpectraMax^®^ M3, Molecular Devices, San Jose, CA, USA). The integrity of the mitochondrial membrane was assessed by osmotically induced volume changes in the mitochondria and spectrophotometric determination of the apparent absorption of the suspension at 540 nm. A mitochondrial suspension (100 μg/mL) was added to the incubation buffer in the absence of respiratory substrates at 37 °C and under constant stirring. The mitochondrial swelling was stimulated with 100 nmol·L^−1^ calcium. The swelling was expressed as a percentage of the absorption of the solution containing mitochondria in the presence of cyclosporin A (0% of mitochondrial swelling) in relation to that absorbed after the addition of FCCP (100% of mitochondrial swelling). For mitochondrial transmembrane potential determination, the probe TMRM (tetramethylrhodamine methyl ester, 400 nmol·L^−1^) was added to the incubation solution containing 100 μg/mL of mitochondria for 1 h prior to the experiment. The transmembrane potential was estimated by fluorescence emitted by TMRM under 580 nm excitation. The transmembrane potential was expressed as the percentage of fluorescence emitted by TMRM-labeled mitochondria in the presence of cyclosporin A (0% of mitochondrial depolarization) relative to that emitted after the addition of FCCP to fully depolarize the mitochondria (100% of mitochondrial depolarization) [23,27,28].

### 4.7. Mitochondrial ROS Concentration

The Amplex Red Hydrogen Peroxide Assay Kit (Life Technologies, Carlsbad, CA, USA) was used to determine mitochondrial ROS concentration. Amplex Red reacts in 1:1 stoichiometry with peroxide in the presence of horseradish peroxidase (HRP) and produces highly fluorescent resorufin 95%. The incubation buffer containing mitochondria was removed from the respiration chamber and immediately supplemented with 50 µmol·L^−1^ Amplex UltraRed reagent and 2 U/mL of Pierce™ Horseradish Peroxidase (HRP, Thermo Fisher, Catalog number: 31491, Waltham, MA, USA). The supernatant was collected after 20 min incubation in the dark. Mitochondrial ROS concentration was determined and compared with H_2_O_2_ standards using a 96-well black plate and a spectrofluorometer (SpectraMax^®^ M3, Molecular Devices, San Jose, CA, USA) at 540 nm emission and 580 nm extinction wavelengths [23,27,28].

### 4.8. Electron Leakage and ATP/ROS Production Ratio

Electron leakage is the loss of the electron from the electron transport chain leading to the formation of superoxide (O_2_^•−^), which is a crucial phenomenon to investigate. In order to compute the percentage of electrons that were leaked from the respiratory chain, the rate of H_2_O_2_ formation must be divided by the rate of mitochondrial O_2_ consumption. In order to ensure accurate results, H_2_O_2_ production and oxygen consumption rates should be expressed using the same units and correspond to the same respiratory state. The measurement of ATP/ROS ratio is necessary to determine the level of ROS production linked to O_2_ consumption. By carrying out these procedures, we were able to precisely determine the degree of electron leakage that was inherently linked to ROS production [23,27,28].

### 4.9. Statistics

Data are presented as the mean ± standard error of the mean (SEM). For graphic and statistical analysis, the software GraphPad Prism 8.4.3 (San Diego, CA, USA) was used. The data distribution was considered normal by the Shapiro–Wilk test. The significant differences in mitochondrial oxygen consumption and the functions were evaluated by the parametric Student *t*-test. *p* < 0.05 was considered statistically significant. This section is not mandatory but can be added to the manuscript if the discussion is unusually long or complex.

## 5. Conclusions

In conclusion, this research offers novel perspectives on how aging affects cardiac mitochondrial function. According to our findings, aging causes a decrease in the mitochondrial respiratory capacity, a decrease in ATP production, an increase in mitochondrial ROS production, and a rise in proton leakage. These aging-related changes in mitochondrial function may be a factor in the emergence of aging-related cardiac diseases and decreased cardiovascular capacity in aged individuals. As a result, further research is required to create novel therapeutic approaches that specifically target mitochondrial dysfunction in aging and age-related diseases.

## Data Availability

The data presented in this study are available on request from the corresponding author.
